# Multiscale morphological analysis of bone microarchitecture around Mg-10Gd implants

**DOI:** 10.1016/j.bioactmat.2023.07.017

**Published:** 2023-08-01

**Authors:** Sandra Sefa, Jonathan Espiritu, Hanna Ćwieka, Imke Greving, Silja Flenner, Olga Will, Susanne Beuer, D.C Florian Wieland, Regine Willumeit-Römer, Berit Zeller-Plumhoff

**Affiliations:** a Institute of Metallic Biomaterials, Helmholtz Zentrum Hereon, Geesthacht, Germany; b Syntellix AG, Hannover, Germany; c Institute of Materials Physics, Helmholtz Zentrum Hereon, Geesthacht, Germany; d Molecular Imaging North Competence Center, Kiel University, Kiel, Germany; e Fraunhofer Institut für Integrierte Systeme und Bauelementetechnologie (IISB), Erlangen, Germany

**Keywords:** Biodegradable magnesium implants, Lacunar-canalicular network (LCN), Vascular porosity, Synchrotron radiation micro computed tomography (SRμCT), Transmission x-ray microscopy (TXM)

## Abstract

The utilization of biodegradable magnesium (Mg)-based implants for restoration of bone function following trauma represents a transformative approach in orthopaedic application. One such alloy, magnesium-10 weight percent gadolinium (Mg-10Gd), has been specifically developed to address the rapid degradation of Mg while enhancing its mechanical properties to promote bone healing. Previous studies have demonstrated that Mg-10Gd exhibits favorable osseointegration; however, it exhibits distinct ultrastructural adaptation in comparison to conventional implants like titanium (Ti). A crucial aspect that remains unexplored is the impact of Mg-10Gd degradation on the bone microarchitecture. To address this, we employed hierarchical three-dimensional imaging using synchrotron radiation in conjunction with image-based finite element modelling. By using the methods outlined, the vascular porosity, lacunar porosity and the lacunar-canaliculi network (LCN) morphology of bone around Mg-10Gd in comparison to Ti in a rat model from 4 weeks to 20 weeks post-implantation was investigated. Our investigation revealed that within our observation period, the degradation of Mg-10Gd implants was associated with significantly lower (p < 0.05) lacunar density in the surrounding bone, compared to Ti. Remarkably, the LCN morphology and the fluid flow analysis did not significantly differ for both implant types. In summary, a more pronounced lower lacunae distribution rather than their morphological changes was detected in the surrounding bone upon the degradation of Mg-10Gd implants. This implies potential disparities in bone remodelling rates when compared to Ti implants. Our findings shed light on the intricate relationship between Mg-10Gd degradation and bone microarchitecture, contributing to a deeper understanding of the implications for successful osseointegration.

## Highlights

• Impact of Mg-10Gd implants on vascular porosity and LCN is assessed in comparison to standard Ti implants for the first time.
• Multiscale imaging and image-based fluid flow simulation were utilized to study the vascular porosity and LCN.
• Significantly lower lacunar distribution was observed in bone around Mg-10Gd compared to Ti implants.

## Graphical abstract

**Figure:**
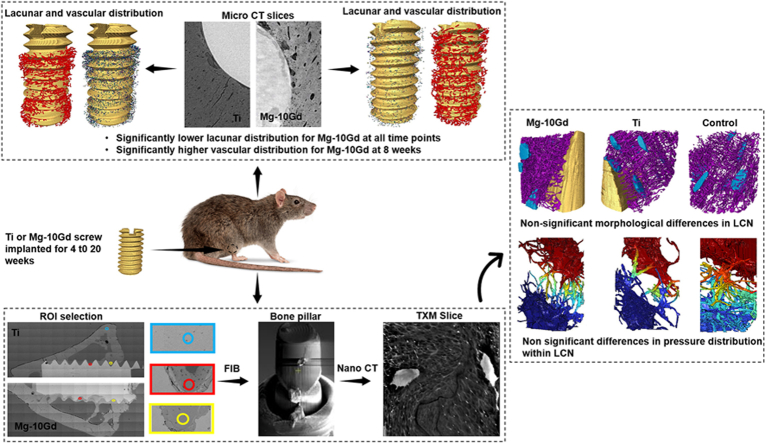

## 1 Introduction

With rapid increase in life expectancy [1] which is concomitant with an aging society, bone is prone to diseases and trauma that can lead to fracture and defects of the skeletal system. This can result in socioeconomic burden and in the worst-case death due to associated comorbidities [[2], [3], [4]]. To curb socioeconomic cost or prevent mortality, the damage caused may require the use of orthopaedic implants to restore bone function. Therefore, the fabrication and integration of bone implants require careful consideration, especially those that degrade over time in the host bone. Bone is a complex and continuously changing tissue whose properties do not only depend on its geometry but also on its microarchitecture. The microarchitecture of bone exhibits a hierarchical organization constituted by three levels of microporosity [5]. The first level is the vascular porosity which consists of the Haversian and Volkmann canals. The second level is the lacunar-canalicular network (LCN) which is associated with the fluid space surrounding the osteocytes and their cell processes. The last level is the collagen-hydroxyapatite porosity which involves the spaces between the crystallites of hydroxyapatite [5]. The different organizational levels form an interconnected network that collectively contributes to the overall transport phenomena within the bone, and consequently its mechanical properties and health. The bone's transport mechanism constitutes the transport of fluids and solutes which are dependent on mechanical strains. Mechanical strains rely on deformations associated with cortical and trabecular bone compressibility which in turn affect osteocyte metabolic function and mechanosensation [6,7]. Osteocytes orchestrate bone remodelling by modulating the recruitment, differentiation, and activity of osteoblasts and osteoclasts to control bone resorption and formation in response to mechanical stimuli and physical damage [8]. Particularly, osteocytes sense mechanical strains as fluid shear stresses and then undergo deformation to directly illicit a response from osteoblasts and osteoclasts *via* mechanical and chemical cross talks. The health of bone tissue majorly depends on efficient fluid and solute transport between its blood supply and cells whereby the vascular porosity serves as low pressure reservoir to relax fluid pressure around osteocytes [9,10]. In addition to mechanosensation and biotransport, osteocytes are hypothesized to be involved in regulating mineral homeostasis by taking advantage of their large surface area presented by the LCN to facilitate rapid mineral exchange [11,12]. It has been shown that lacunar density depends on the microanatomical bone type, i.e. cortical or trabecular bone, and age [[13], [14], [15]], as well as loading status [16,17]. Notably, in cortical bone, lacunar volume and surface area have been shown to be between 31% and 43% higher compared to that of trabecular bone [18].

During orthopaedic interventions, significant micro damage in the form of bone cell apoptosis and disruption of transportation pathways occur in the host bone as far as 1-2 mm from the implantation site [19]. This results in extensive remodelling of the microarchitecture in the host bone and the formation of woven bone at the peri-implant interface which is sequentially replaced by matured lamellar bone as healing progresses. Restoration of bone function after trauma requires an extensive understanding of the structural and functional adaptation of biotransport mechanisms in its microarchitecture. Past studies based on 2D scanning electron microscopy suggest that the early phase of peri-implant bone healing is characterized by a higher osteocyte density within the bone around microrough Ti [20] and Ti6Al4V [20,21] implants. Furthermore, microrough Ti implants have been shown to retain morphologically round lacunae [22] with increasing LCN complexity over time [23]. Shah et al. further reported that osteocytes directly attach to Ti6Al4V implants over time and retained between 38% and 42% more canaliculi per lacunar surface area [21]. Regarding bone vasculature, Palmquist et al. showed that blood vessels remain close to LCN within the bone matrix [24]. As neovascularization is indispensable to osteogenesis, it has been shown that changes in implant topology affects the pattern of vascularization which in turn affects the fluid flow and the nourishment of bone cells [25]. While extensive knowledge exists on the interaction of permanent implants with bone microstructure, the impact of biodegradable magnesium (Mg)-based implants and the resulting changes in local chemistry on the microarchitecture of bone is lacking.

The potential use of Mg-based implants in orthopaedics has multiple benefits over conventional permanent implants. To begin with, Mg is biodegradable thus requiring no secondary surgery for implant removal, reducing cost and further patient comorbidity [26]. In comparison to permanent implant materials such as Ti, Mg implants have a Young's modulus more similar to bone thus reducing stress shielding effects. Stress shielding negatively affects bone remodelling and can even lead to osteoporosis in the worst-case scenario [27]. As pure Mg often degrades too rapidly, it is alloyed with other metals such as gadolinium (Gd) to tailor its degradation rate. In Mg-based implant design, the addition of 10 weight percent (wt.%) Gd to form Mg-10Gd has been shown to enhance its corrosion resistance and mechanical properties. More specifically, the incorporation of Gd into Mg improves its ductility and reduces its anisotropy through texture weakening and slip activation of Mg matrix. By this, the directional dependency of the material properties is mitigated resulting in robust resistance against mechanical failure [28,29]. Particularly noteworthy are the effects of solid solution strengthening observed in Mg-10Gd alloys consequently resulting in improved tensile yield strength (120–240 MPa), ultimate strength (250–450 MPa) and 12–30% elongation [29]. The degradation rates of Mg-10Gd implants have been studied before, showing a homogenous degradation *in vivo* [30] with reported degradation rates between 0.15 and 1.15 mm/year [[30], [31], [32]]. For the samples used in this study, the degradation rates are 0.6 mm/year, 0.4 mm/year and 0.3 mm/year at 4, 8 and 12 weeks respectively [30]. Despite the current usage of Gd as a contrast agent in the clinic, concerns regarding its potential toxicity persist. The materials utilized in this study have demonstrated a non-toxic nature when evaluated in cell culture settings [30,33]. Recent reports have provided evidence that the Gd content in Mg-10Gd remains confined to the degradation layer of the implant, affirming that Gd does not migrate to other tissues during the degradation process [[34], [35], [36]]. Thus, Mg-10Gd alloys emerge as a viable option for temporary implant materials [37,38].

The interaction between Mg-10Gd alloys and bone has been studied at several length scales. At the tissue level, studies have shown that Mg-10Gd yields good bone volume fraction with good bone-implant contact [30,34] and low degradation rates [30]. Also, nanoscale information has revealed that the ultrastructure of bone around Mg-xGd (x = 5 and 10 wt.%) implants is different from that of Ti in terms of hydroxyapatite platelet (HAP) crystal size and lattice spacing with possible deposition of Mg in the bone matrix [39]. Generally, Grünewald et al. reported an alteration in the ultrastructure of bone associated with Mg-based implant degradation [40] in addition to reports by Meichel et al. further confirming that Mg stimulates localized adaptation of the bone ultrastructure [41,42].

Up to date, a knowledge gap between ultrastructure and microstructure information remains, as information on the impact of Mg degradation on the LCN is scarce. So far, one pilot study has indicated that different degradation rates of Mg-based implants may yield different LCN organization [43]. In this study, which is in part a continuation of that of Krüger et al. [30], we study the microarchitecture and porosity of the interfacial bone of Mg-10Gd in comparison to Ti across the micro- and nanoscale using 3D synchrotron radiation-based computed tomography. We hypothesize that the degradation of Mg-10Gd will alter the chemical and mechanical environment perceived by the osteocytes which will affect mechanosensation, biotransport and mineral homeostasis mechanism within the bone. Our approach is to first investigate the impact of Mg-10Gd degradation on the vascular and lacunar porosity in comparison Ti at 4, 8 and 12 weeks post-implantation using synchrotron radiation-based micro computed tomography (SRμCT). The use of SRμCT enables the study of the bone microarchitecture at a resolution of a few micrometers [44] revealing subtle information due to an excellent signal-to-noise ratio. Secondly, we study the LCN morphology of the interfacial bone of Mg-10Gd in comparison to Ti at 10 and 20 weeks post-implantation using 3D synchrotron radiation-based transmission X-ray microscopy (TXM). With TXM, the morphological changes in the LCN can be studied non-destructively in detail [45]. With the incorporation of Zernike-based phase contrast in the image acquisition set-up, low contrast components in the bone tissue can be distinguished [45,46]. Finally, image-based finite element modelling is employed following TXM measurements to model the fluid flow within the LCN within the interfacial bone of Mg-10Gd in comparison to Ti. This hierarchical approach to combine information from two imaging length scales in conjunction with image-based fluid flow simulation will provide in-depth knowledge on the adaptation of bone in the presence of Mg degradation, bridging the knowledge gap between the tissue level and the ultrastructural level.

## 2 Materials and method

### 2.1 Material production

The detailed process of the production of Mg-10Gd screws used in this study can be found here [47,48]. Briefly, the first step was melting and casting of Mg using permanent mould direct chill casting technique. 10 wt.% of Gd was added to the molten Mg at 700 °C. The molten materials were poured into a permanent steel mould and solution heat treated (T4) followed by indirect extrusion with an extrusion ratio of 84 to the final diameter of 12 mm. Wire erosion was employed to cut the rods into smaller half sections consisting of 3 mm diameter each. Finally, the screw slit head was formed by milling and their shape by machining (length 4 mm, diameter 2 mm, thread M2 and a 0.5 × 0.5 mm slotted screw head). The screws were cleaned in an ethanol bath, packed into individual tubes and were then sterilized with Gamma radiation with a dose of 27 kG [49]. The Ti screws, used as reference materials, were purchased from Prominic AB (Mölndal, Sweden). The Ti screws were autoclaved after they were cleaned in ethanol bath, dried and packed in glass vials.

### 2.2 Animal experiments

For the SRμCT study, thirty explants (bone blocks with implant screws) obtained at 4, 8 and 12 weeks post-implantation from a previous animal experiment [30] involving Sprague Dawley male adult rats were used. The Malmö/Lund regional board for animal research, Swedish Board of Agriculture approved the animal experiment with approval number DNR M 188-15. Details of the implantation protocol have been earlier noted [30].

The TXM animal experiment involved eighteen Sprague Dawley rats obtained from Charles River Laboratories (Sulzfeld, Germany). The animal experiment was approved by the Ethics Committee for Animal Experiments at Christian-Albrechts University of Kiel, Germany with approval number V 241-26850/2017(74-6/17). The animals were housed in cages with two or three animals per cage and fed ad libitum. In brief, the rats were administered a general anaesthesia with an intraperitoneal dose of 75 mg/kg Ketamine and 0.5 mg/kg Medetomidine. The legs of the rats were shaved and disinfected with a skin disinfectant (Octenisept) and a longitudinal incision was made medially along the lower leg 8 mm below the knee joint (about 2 mm distal to the knee joint and about 1 mm median to the crest of the tibia) with a scalpel. After exposing the tibia metaphysis, an osteotomy was created by drilling with a 1.4 mm electric drill (dental micromotor Marathon s04 (N7S)). The screws were inserted monocortically using a manual hand drill, with about two or three threads of the screw sticking out of the tibial plate. Each rat received an Mg-10Gd screw or a Ti screw making a total of eighteen screws with random allocation to the left or right leg. Afterwards, the wound was sutured with an absorbable suture (5-0, Vicryl USP) and the rats were placed on a warm plate and partially antagonized (Atipamezole, 1 mg/kg bw, 50 μL subcutaneously). The rats were administered Metamizole (100 mg/kg) after 2 h of surgery and tramadol for 5 days after surgery via drinking water (in the dosage of 0.5 mg/ml drinking water, according to Lang et al. [50]. After 72 h, the rats received an analgesic dose of 0.5 mg/mL of tramadol *via* drinking water. The rats were fed ad libitum and moved freely in the cage. The animals were euthanized at 10 and 20 weeks post-implantation with a lethal dose of anaesthetic. The legs were dissected to expose the bone around the implant area. The bone surrounding the implants was explanted using a trephine bur of 6 mm diameter. The bone-implant blocks were then fixed with 70% ethanol for at least 1 day followed by dehydration in graded series of ethanol and embedded in methyl-methacrylate based resin (Technovit 7200, VLC; Hereaeus Kulzler, Germany) by LLS Rowiak GmbH (Hannover Germany). Summary of the number of explants and time points of SRμCT and TXM study are in Table 1.

**Table 1:** Number of explants investigated via SRμCT and TXM.

	SRμCT			TXM	
Time point (weeks)	4	8	12	10	20
Mg-10Gd explants	5	5	5	4	5
Ti explants	5	5	5	4	5

### 2.3 Image acquisition and analysis for SRμCT

#### 2.3.1 SRμCT image acquisition

As previously stated, explants of rat tibia obtained at 4, 8 and 12 weeks post-implantation were used for the SRμCT study. The SRμCT image data acquisition have been described in detail in our previous study [30]. In brief, the image acquisition was performed at the P05 imaging beamline (IBL) of the PETRA III storage ring at the Deutsches Elektronen Synchrotron (DESY), which is operated by Helmholtz-Zentrum Hereon Geesthacht, Germany [51,52]. The samples were scanned in absorption contrast mode at an energy between 25 and 45 keV, depending on the implant material, at a stepwise rotation with 1200 projections. The tomographic reconstruction pipeline for microtomography data at the IBL [53] in MATLAB (R2021b MathWorks, Inc., Massachusetts, USA) was used to reconstruct the tomograms based on the filtered back projection (FBP) algorithm using the ASTRA back projection toolbox [54,55]. The images were resampled to a voxel size of 5 μm for the analysis.

#### 2.3.2 SRμCT image processing and analysis

**Fig. 1:**
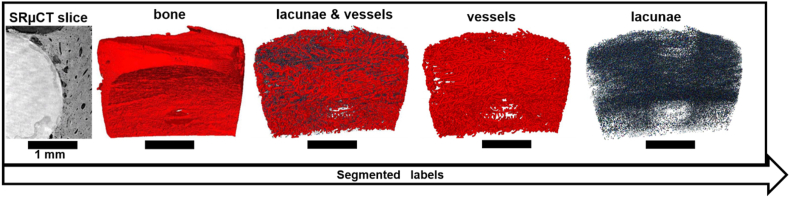
Original SRμCT cross-section and volume renderings of segmented labels lacunae and vascular porosities.

The processing of the SRμCT data have been described by Küger et al. [30]. Prior to segmentation of the lacunar and vascular porosity, first, the bone tissue and the implant were automatically segmented using a U-Net convolutional neural network [56,57]. Employing the segmentation method developed by Nunez et al. [58], the bone porosities (lacunar and vascular porosity) were extracted from the bone label. In brief, the bone labels were cleaned using a 3D opening operation. In the second step, interactive thresholding was performed to segment the bone porosity from the bone. To remove the clustered voxels that should be a part of the background but were incorrectly identified as bone porosity, a third step was performed to generate a bone mask. To this end a 3D closing operation was performed on the clean bone to fill in the bone porosity. In order to extract only the bone porosity, the bone porosity obtained from the second step was inverted and combined with the solid bone mask in an ‘AND’ logical operation resulting in a data set containing only the bone porosity. Finally, the lacuna porosity was separated from the vascular porosity *via* component labelling and the vascular porosity was skeletonized and subsequently analysed in MATLAB (R2021b MathWorks, Inc., Massachusetts, USA). Segmented labels are shown in Fig. 1.

To understand the local effect of a degrading Mg-based implant on the interfacial bone porosity, all quantified parameters were obtained within a 200 μm volume of interest (VOI), which represent the regions between the screw thread and screw tips. The 200 μm VOI was generated by dilating the screw (composed of residual metal plus degradation layer) 40 times. The quantified parameters are defined as: vascular number density (N.Vs) and lacunar number density (N.Lc). All quantified parameters were normalized by the total bone volume (BV) (bone plus pores) which was calculated using equation (1). Lacunar and vascular number density were calculated as the total number of objects normalized by BV (equation (2)).

(1) BoneVolume=Sumofvoxelswithinbonemask×voxelsizecubed

(2) $$Lacunar/Vascular\;number\;density=\frac{Number\;of\;lacunae\;or\;vessels}{BV}$$

### 2.4 Image acquisition and analysis for TXM

#### 2.4.1 Scanning electron microscope (SEM) image acquisition and analysis

The embedded explants from the second animal experiment at 10 and 20 weeks were cut into two halves using a diamond saw (Well Diamond Wire Saws SA, Mannheim, Germany) to expose the bone and the implanted screws. The samples were then cleaned with ethanol and polished using Diamond suspension (particle size 3 μm) for 7 min and viewed under a light microscope to ascertain effective polishing. The polished samples were sputtered with gold for a minute using Cressington sputter coater (TESCAN GmbH, Dortmund, Germany). Afterwards, the samples were scanned using TESCAN AMBER X (TESCAN GmbH, Dortmund, Germany) scanning electron microscope (SEM) with a current of 10 pA and a voltage of 10 kV. The magnification was set to yield an image with a pixel size of 0.195 μm.

#### 2.4.2 Sample preparation for TXM specimens

**Fig. 2:**
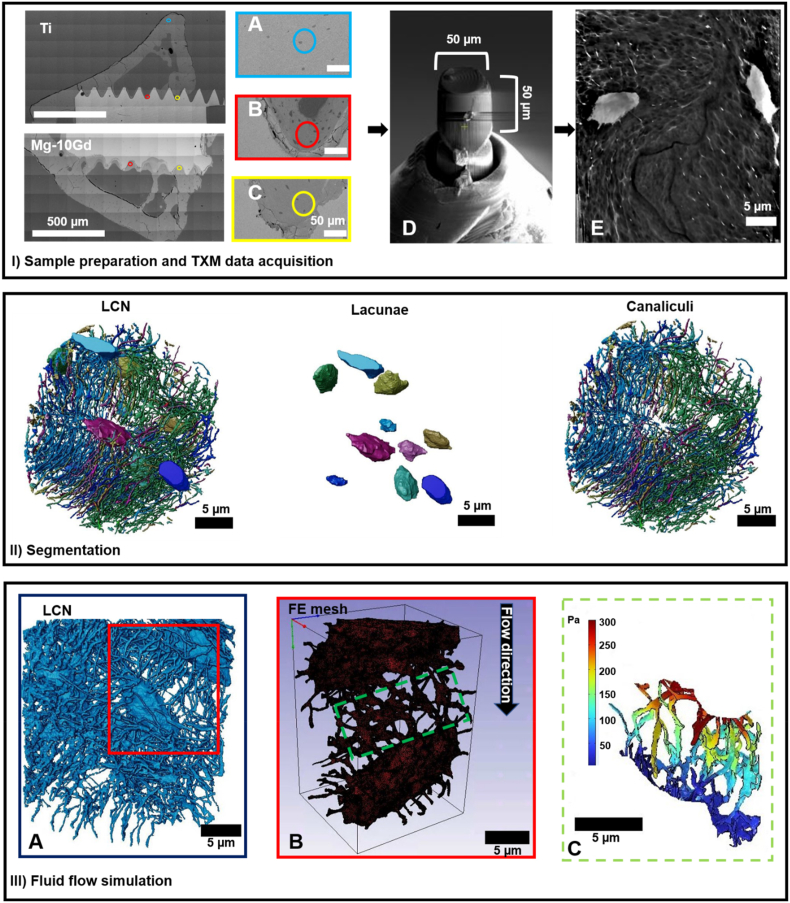
Sample preparation steps for TXM image data acquisition and finite element modelling. (Panel IA) – (Panel IC) SEM slices showing the selection of ROIs; (Panel IA) ROI from a region far from Ti implant to serve as control. (Panel IB) ROI from trabecular region obtained from either Ti or Mg10Gd. (Panel IC) ROI from cortical region obtained from either Ti or Mg10Gd. (Panel ID) bone pillar for TXM experiment produced by FIB-milling, imaged using FIB-SEM. (Panel IE) example slice through 3D TXM image data. (Panel II) is a volume rendering of segmented TXM image data into LCN, consisting of lacunae and canaliculi (the colours represent the labelling of the LCN). (Panel IIIA) – (Panel IIIC) represents finite element simulation of the selected lacunae pair; (Panel IIIA) shows selection of lacunae pair, (Panel IIIB) shows meshing of lacunae pair, (IIIC) represents selected ROI for quantitative analysis.

From the SEM image of each sample (Fig. 2 panel I), two region of interests (ROIs) were defined for each sample of each implant material per time point: one from the trabecular region (Fig. 2 panel IB) and one from the cortical region of the bone implant interface (Fig. 2 panel IC) in order to obtain representative samples across the bone. Bone specimens were also cut from bone in Ti samples far from the implantation area (1 mm) to serve as an additional control (Fig. 2 panel IA). The ROIs were then cut using focused ion beam (FIB, Helios G4 PFIB CXe, Thermo Fisher Scientific) milling (Fraunhofer Institut für Integrierte Systeme und Bauelementetechnologie (IISB), Erlangen, Germany) (Fig. 2 panel ID). Overall, 45 pillars (Table 2) from bone specimen with a diameter approx. 50 μm and a height of 50 μm were milled and mounted on custom-made pins (Fig. 2 panel ID).

**Table 2:** Table B: Bone specimens used for TXM investigation.

	Cortical bone		Trabecular bone	
Time Point (Weeks)	10	20	10	20
Mg-10Gd bone pillars	4	5	4	5
Ti bone pillars	4	5	4	5
Control (bone from Ti samples far from the implantation area)	4	5	-	-

#### 2.4.3 TXM image acquisition

The bone pillars were imaged using TXM at the P05 nanotomographic endstation, PETRA III, DESY, which is operated by Hemholtz-Zentrum Hereon [59]. The imaging was performed using a monochromatic beam with an energy of 11.1 keV which was selected using a Si111 double crystal monochromator (ΔE/E ∼ 10^−4^). The illumination of the bone specimens was performed using a beam shaping condenser with square fields of 50 μm × 50 μm and a Fresnel zone plate objective lens with an outermost zone width of 50 nm. To achieve phase contrast, Zernike phase rings were placed in the back focal plane of the Fresnel zone plate. The X-ray Optics and Applications group of the Paul-Scherrer-Institut (Switzerland) designed and fabricated all X-ray optics. A Hamamatsu C12849-101U camera with a sCMOS chip with 6.5 μm physical pixel size and a 10-μm Gadox scintillation layer was used and placed roughly 20 m behind the sample. The high resolution scanning generated 3D images with 22.8 nm voxel size. The raw image data were reconstructed using the P05 reconstruction pipeline for nanotomography data based on TomoPy [60] with a binning factor of two, resulting in a voxel size of 45.6 nm. A slice through the 3D TXM image data is shown in Fig. 2 panel IE.

#### 2.4.4 TXM image processing and analysis

##### 2.4.4.1 Image processing

The high-resolution image data were first filtered using an iterative non-local mean filter to remove textural noise [61] caused by back projection during image data acquisition, followed by segmentation in Avizo (version 2021.1, Thermo Fisher Scientific, Waltham, USA). Interactive thresholding was employed to separate the LCN (Fig. 2 panel II) from the ECM followed by manual corrections. To extract the lacunae from the LCN, an opening operation was performed to remove the canaliculi retaining only the lacunae (Fig. 2 panel II). To extract the canaliculi from the LCN, the lacunae were inverted and combined with the LCN using a Boolean ‘AND’ operation (Fig. 2 panel II).

##### 2.4.4.2 Quantification of lacunar descriptors, canalicular descriptors & LCN porosity

The quantification of the lacunar and canalicular morphological descriptors was performed using the label analysis module in Avizo (Version 2021.1, Thermo Fisher Scientific, Waltham, USA). The lacunar size, which is constituted by the lacunar volume (Lc.V) and surface area (Lc.SA) was quantified. The shape of the lacunae was quantified by calculating the sphericity and smoothness (compactness) using (3), (4):

(3) $$Sphericity=\frac{\pi^{\frac{1}{3}}{\left(6\times Volume\right)}^{\frac{2}{3}}}{Surface\;area}$$

(4) $$Smoothness=\frac{{Surface\;area}^{3}}{36\times \pi \times {Volume}^{2}}$$

The surface area (Ca.SA) and volume (Ca.V) of the canaliculi were also quantified. For all the aforementioned parameters, the mean value per investigated ROI was obtained. Additionally, the number of the canaliculi per lacuna surface area or canaliculi areal density (N.Ca/Lc.SA) and the canaliculi junction density (Ca.nodes/BV) were also quantified using the auto skeleton module in Avizo (version 2021.1, Thermo Fisher Scientific, Waltham, USA). The LCN porosity (LCN/BV) was quantified as the ratio of the sum of voxels within the LCN labels to the sum of voxels within the entire bone label.

##### 2.4.4.3 Distance distribution of lacunae and canaliculi

To explore the spatial arrangement of the lacunae and their canaliculi within the bone matrix their distance maps were calculated [62,63]. The distance map depicts the distance of the bone matrix from the nearest lacunar and canalicular. For this, the 3D distance maps of the lacunae and canaliculi were first calculated. In the second step, the normalized cumulative histogram of the distance maps was calculated. The values which correspond to 50% of the distribution, denoted as Lc.Dist_50_ and Ca.Dist_50_ were determined for lacunae and canaliculi respectively. The resulting values indicate that 50% of the bone matrix can be found within a distance Lc.Dist_50_ from the nearest lacunae and Ca.Dist_50_ from the nearest canaliculi respectively.

#### 2.4.5 Image-based computational fluid dynamics analysis

##### 2.4.5.1 Model generation

To further understand the impact of degradable Mg-10Gd implants on the functionality of the LCN, image-based fluid flow simulations were performed. To this end, a sub-volume consisting of a pair of lacunae with their connected canaliculi was extracted from the segmented LCN of each bone specimen for both Mg-10Gd, Ti and the control bone specimens (Fig. 2 panel IIIA). The extracted sub-volumes (lacunae pairs) were modelled to represent the ‘basic unit’ of the LCN within which cellular communication occurs. The lacuna pairs were meshed using Simpleware ScanIP (version P-2019.09, Synopsis, North Carolina, United States) voxel-meshing software to create tetrahedral elements. The meshed lacuna pairs (Fig. 2 panel IIIB) were then exported to COMSOL Multiphysics (version 6.0, COMSOL AB, Stockholm, Sweden) for the fluid flow simulation implementation.

##### 2.4.5.2 Fluid flow simulation

The fluid flow simulation used in this study was adapted from previous work [64,65]. Darcy's Law [65] (equation (5)) was employed to investigate the pressure and velocity distributions of interstitial fluid within the LCN pair, i.e.:

(5) $$u=-\frac{k}{\mu }\nabla p$$

where u is Darcy's velocity, k is the permeability of the porous media, μ is the fluid's dynamic viscosity, ∇ p is the load induced fluid pressure gradient.

The permeability and porosity of the generated mesh of the lacunae pairs as well as the density and dynamic viscosity of the simulated interstitial fluid were considered. All the parameters used for the fluid flow simulations are summarized in Table 3. During mechanical loading in bone, a pressure gradient is generated from the compression and tension which occur in different regions of the bone to drive fluid across the bone network [[66], [67], [68]]. This phenomenon was incorporated into the model by applying a pressure gradient across the model. Thus, an inlet pressure of 300 Pa was assigned to the inlet on one face and the opposite face was assigned an outlet pressure of 0 Pa in the flow direction [[66], [67], [68]]. The remaining faces of the mesh were assigned symmetry boundary conditions. The resulting stationary fluid equations were solved and the average pressure and velocity were analysed for the canaliculi that connect the lacuna pair (Fig. 2 panel IIIC). To this end, the connected canaliculi were segmented from the lacunae pair and multiplied with the pressure and velocity matrix obtained from the solution to the fluid flow equations in MATLAB (R2021b MathWorks, Inc., Massachusetts, USA). To account for differences in the volume of the analysed region, the average pressure and velocity were further normalized by the total volume of the connecting canaliculi.

**Table 3:** Parameters used for Finite element modelling.

Parameter	Value	Unit
Permeability [65]	10^–18^	m^2^
Porosity [65]	5	%
Density of salt water [69]	997	kg∙m^−3^
Dynamic viscosity [70]	8.55 × 10^−4^	kg∙m^−1^∙s^−1^

### 2.5 Statistical analysis

All statistical analysis was performed in Origin 2021b (OriginLab Northampton Massachusetts, USA). The normality of the data was first evaluated using Shapiro Wilk normality test. Using one-way analysis of variance (ANOVA), the means of all quantified parameters were evaluated between each material and time point. Also, to quantitatively evaluate the impact of Mg-10Gd on the specific bone types, group means were tested between the trabecular and cortical bone specimens. To explicitly determine the significance of the parameters among the groups, a post hoc analysis for pairwise comparison among means was performed using Tukey's honestly significant difference (HSD) tests. A significance level of p < 0.05 was considered significant.

## 3 Results and discussion

### 3.1 Lacunar and vascular porosity distribution based on SRμCT analysis

**Fig. 3:**
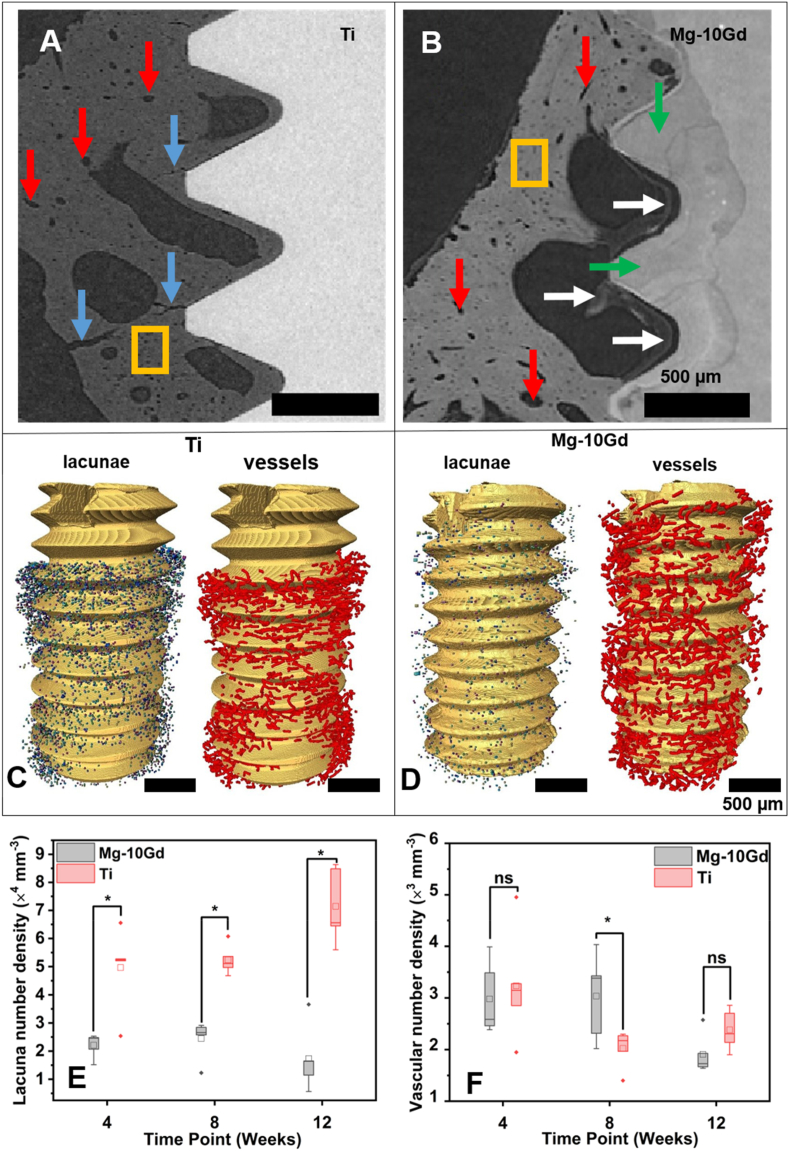
(A–B) are 2D SRμCT slices showing the bone around Ti implant (A) and Mg-10Gd implant (B). The red arrows show vascular pores, yellow boxes show lacunae, blue arrows show cracks in the bone around Ti, white arrows show unmineralized bone regions around Mg-10Gd and green arrows show Mg-10Gd degradation layer. (C) 3D visualization of lacunar distribution (colored dots) and vascular distribution (red) around Ti screw. (D) Visualization of lacunar distribution (colored dots) and vascular distribution (red) around Mg-10Gd screws. (A–D) were obtained at 8 weeks post-implantation. (E & F) are boxplots of the lacunar and vascular distributions around Mg-10Gd and Ti implant screws. ns means not significant and * means significant (p < 0.05). Within each boxplot, the horizontal line represents the median while the small box represents the mean.

Fig. 3A-**3D** show the 2D and 3D visualization of the lacunar and vascular porosity distributions between biodegradable Mg-10Gd and Ti at the bone implant interface (200 μm VOI) at 8 weeks. It appears that the lacunar porosity around Ti is denser in comparison to Mg-10Gd which displays sparsely distributed lacunae around the implant Fig. 3C & D. Quantitatively, we found a significantly (p < 0.05) lower lacunar number density at all time points around Mg-10Gd in comparison to Ti (Fig. 3E). The vascular number density was significantly higher at 8 weeks post-implantation (p < 0.05) for Mg-10Gd compared to Ti but not at 4 and 12 weeks (Fig. 3F). The descriptive statistics for the lacunar and vascular porosity distributions are summarized in Table S1 in the “Supporting Information”. Our results may imply that pressure regulation and metabolic transport within the interfacial bone around Mg-10Gd at 4 and 12 weeks were similar to that of Ti but were significantly enhanced within Mg-10Gd at the intermediate time point (8 weeks) (Fig. 3C, D & 3F). Although it is not clear what might account for the significantly high vascular density within the interfacial bone of Mg-10Gd at 8 weeks, it may be conceived that there was a high demand to regulate Mg homeostasis thus the need for enhanced circulation. However, this is not corroborated by the volume loss measurements of the samples used in this study, where the relative volume loss between the time of implantation and 4 weeks of healing is higher than that between 4 and 8 weeks [30]. Furthermore, Tartrate resistant acid phosphatase (TRAP)-positive area percentages (TRAP%) analysis of the same samples in Krüger et al. [30] showed a continuous decline in TRAP-positives regions for Ti from 4 to 12 weeks meanwhile that of Mg-10Gd decreased from 4 to 8 weeks and increased again slightly from 8 to 12 weeks. TRAP-positive regions represent osteoclasts activated for bone resorption. It is hypothesized that osteoclasts stimulate angiogenesis by the secretion of osteopontin [71] or Metalloproteinase-9 [72]. Remarkably, at every bone remodelling compartment, there is a close proximity between osteoclasts and blood vessels facilitating the efficient exchange of signals and molecules between these two entities [72]. However, the TRAP % analysis was insufficient to elucidate the underlying cause of the observed increased vascular porosities observed around Mg-10Gd at 8 weeks. To gain deeper insights and refine our understanding of the observed phenomena, further research is necessary. This could involve exploring alternative markers or analytical methods that provide a more comprehensive assessment of angiogenesis, cytokine signaling, or cellular interactions. The modulation of pressure within the LCN by the vascular porosity is crucial for the efficient exchange of pore fluid between the two porosity levels [73]. Previous studies have shown that Mg accumulates around blood vessels and the bone marrow for moderate degradation speeds [40]. Iskhakova et al. have also reported that Mg deposits around blood vessels involving the samples used in this study [36].

In this study, we observed significantly lower lacunar number density around Mg-10Gd implants compared to Ti implants throughout the observed time points (Fig. 3C–E). We hypothesize that there could be two potential factors contributing to the observed results: either there was an enhanced bone formation specifically around Ti implants or the degradation of Mg-10Gd had an impact on the bone formation process. The increased bone formation observed around Ti implants could be attributed to two potential factors. Firstly, it could be linked to a higher occurrence of micro cracks (Fig. 3A), which might have stimulated bone remodelling [74]. Secondly, it is probable that the osteoblastic activities prior to differentiation were enhanced, leading to accelerated bone formation in the vicinity of Ti implants. A high lacunar distribution is commonly interpreted as a marker for high bone turnover rate [75,76] and bone mass [77]. Hence, the higher lacunar density surrounding Ti implants may imply higher degree of bone remodelling compared to Mg-10Gd. This may explain the significantly higher bone volume fraction for Ti implants in comparison to Mg-10Gd implants at the 4 and 8 weeks observed by Krüger et al. in the same samples [30]. Specifically, the recorded bone volume fraction (BV/TV) for Mg-10Gd were 25.1 ± 8.5%, 34.9 ± 5.0% and 47.6 ± 6.2% respectively while that of Ti were 48.8 ± 0.4%, 51.3 ± 4.6% and 49.1 ± 7.7% at 4, 8 and 12 weeks respectively [30]. By considering the connection between bone mass and osteocyte density [75,76] it became apparent that the higher BV/TV observed for Ti may contribute to the higher number of osteocytes present in comparison to Mg-10Gd. Lower BV/TV implicates lower overall volume of bone tissue available for osteocyte occupancy. Although Mg-based implants are generally considered osteogenic [37,78,79], the result from the current study in conjunction with that of [30] points to a low bone formation around Mg-10Gd implants during bone healing. As osteoblasts differentiate into osteocytes, any factors or conditions that interfere with osteoblast activities could influence the subsequent osteocyte population [80,81]. Perturbations in osteoblast function, such as reduced proliferation, impaired mineralization, compromised signaling pathways or extracellular matrix stiffness can affect the number and viability of osteoblasts that successfully transition into osteocytes [80,81] thus impacting remodelling and overall health. The impact of Mg on osteoblasts has been a subject of scientific investigation, with contradictory reports emerging from various studies. He et al. and Burmester et al. first reported an upregulation of osteoblast marker such as ALPL, BSPII and osteocalcin in cell lines in the presence of Mg ions [82,83]. Later, several other researchers reported contradictory finding pointing to the direction that Mg inhibits osteoblastic activity [[84], [85], [86], [87]]. The contradictions may arise from several factors such as variations in study models, experimental protocols, Mg concentrations and alloys used. It could also be hypothesized that the incorporation of Mg degradation products in the bone altered the bone matrix mineral content and therefore the chemical stimuli sensed by the osteocytes in the vicinity of Mg-10Gd implants because of the mutual dependence of the osteocytes and the extracellular matrix. As confirmed by several researchers, Mg has been shown to be distributed in the lacunar network upon degradation [40] and directly influences the nano and crystal structure at the bone implant interface in terms of structure, particle size, orientation [40,88] and composition [35]. Peruzzi et al. revealed that Mg-10Gd degradation affects the bone mineral content in its vicinity where lower levels of Ca and P were detected around Mg-10Gd implants in comparison to Ti [35]. In Krüger et al. [30], where the degradation rate of the same samples used in this study was reported to be below 1 mm/year, unmineralized interfacial bone regions could be observed from SRμCT analysis after 8 weeks of implantation (See Fig. 3B in current study and Figure 9 in Krüger et al. [30]) possibly indicating the alteration of bone matrix mineral content due to the incorporation of Mg within the bone. The lower remodelling rate of the bone around Mg-10Gd could explain why Zeller-Plumhoff et al. recorded lower HAP spacing and size around Mg-10Gd in comparison to Ti [39]. Several other reports relating to higher osteocyte density around permanent bone implants have been presented [20,21,89] particularly that of Shah et al. reported enhanced bone maturation and retention of higher density of less aged osteocytes on Ti6Al4V implant surface [21]. The adaptability of bone tissue around Ti-based implants has been well established [21,90] and known to be osteogenic [91]. It has been shown that Ti-based implants form an oxide layer which upregulates ALP activities indicative of enhanced degree of osteoblast differentiation [92]. The observed distribution pattern of lacunae in this study corroborates previous research that has reported increased density of osteocyte populations surrounding Ti implants [21,90]. The significantly lower vascular density around Ti implants at 8 weeks warrants further research to elucidate the underlying mechanism for this outcome.

### 3.2 Evaluation of the LCN architecture based on TXM data

#### 3.2.1 LCN organization and morphology within interfacial bone of Mg-10Gd, Ti and the control bone specimens

**Fig. 4:**
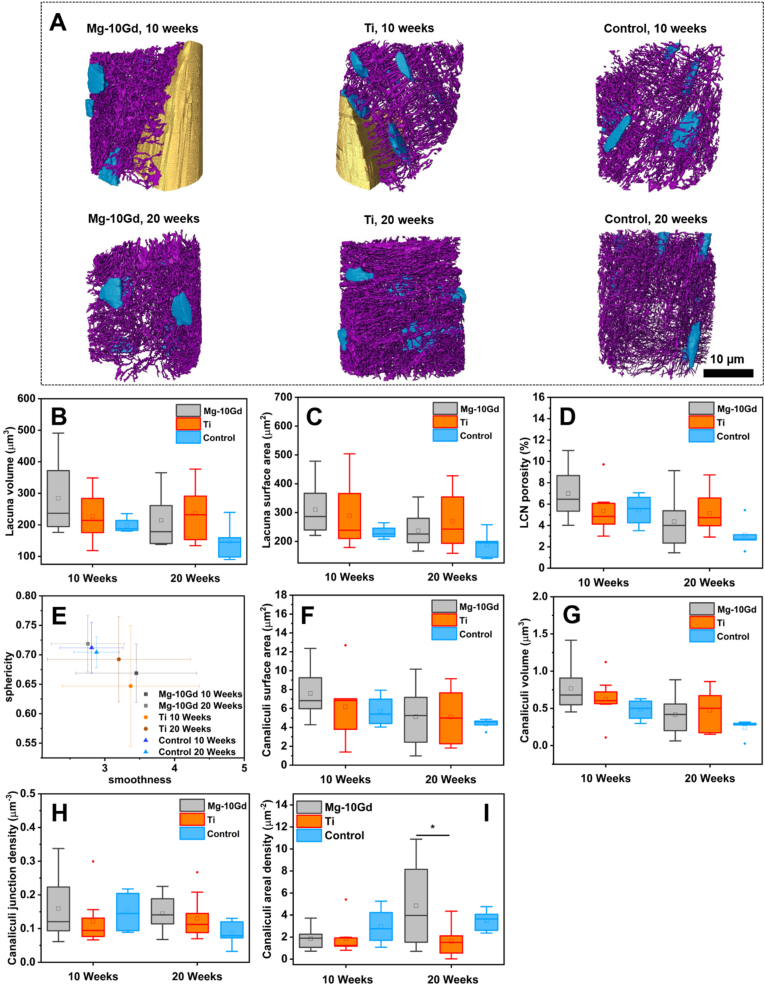
**(A)** 3D rendering of the TXM image data showing the organization of the LCN (lacunae are blue, canaliculi are purple and screws are yellow) in the interfacial bone of Mg-10Gd, Ti and within the control at 10 and 20 weeks. The canaliculi of Mg-10Gd appeared to have enlarged surface area than that of Ti and the control at 10 weeks but at 20 weeks, the canaliculi surface area appeared similar for both implants and the control bone specimens. **(B**)–(I**)** Boxplot of lacunar and canalicular descriptors at 10 and 20 weeks post-implantation: **(B)** lacunar volume, **(C)** lacunar surface area, **(D)** LCN porosity and **(E)** lacunar shape descriptor, (**F)** canalicular surface area, **(G)** canalicular volume, **(H)** canalicular junction density **(**I**)** canalicular areal density. * means significant (p < 0.05). Within each boxplot, the horizontal line represents the median while the square box represents the mean.

Conventional lacunar size and shape parameters have been calculated in this study and are consistent with previous reports ranging from 100 μm^3^ to 500 μm^3^ for lacunar volume [62,93] and lacunar sphericity ranging from 0.71 to 0.73 in shape [18]. The organization of the 10.13039/501100001941LCN is displayed in Fig. 4A for representative samples and the morphological evaluations of the lacunar descriptors are plotted as boxplots in Fig. 4B-**E.** The summary of the descriptive statistics is found in Tables S2A and 2B in the “Supporting Information”. Generally, the mean lacunar volume, surface area and the LCN porosity appeared higher for Mg-10Gd than Ti and the control specimens at 10 weeks but the order was reversed at 20 weeks, where the lacunae around Ti had higher mean volume, surface area, and LCN porosity (Fig. 4B-**D**). Furthermore, the mean lacunar volume, surface area and LCN porosity declined over time for Mg-10Gd and the control but not for Ti (Fig. 4B-**D**). The observed differences were not significant. There is evidence that the osteocytes within the bone adjacent to the implants were larger in size than those in the control bone which indicates a less aged and woven bone morphology near the implant [21] compared to mature bone in the control [94]. However, the observed difference did not reach the level of statistical significance. For the lacunar shape (Fig. 4E), at 10 weeks, the highest sphericity index (0.71 ± 0.04) was associated with the lacunae of the control bone specimen followed by that of Mg-10Gd (0.67 ± 0.05) and finally that of Ti (0.65 ± 0.10). At 20 weeks, the highest sphericity index was recorded for the lacunae around Mg-10Gd (0.72 ± 0.05) followed by that of the control (0.70 ± 0.03) and finally Ti (0.69 ± 0.08). Regarding the smoothness, at 10 weeks, a value of (3.37 ± 0.98) was recorded for the lacunae around Ti, (3.45 ± 0.87) for Mg-10Gd and finally that of the control bone specimens was (2.81 ± 0.45). At 20 weeks, Ti had the highest smoothness (3.20 ± 1.02) followed by Mg-10Gd and lastly the control (2.88 ± 0.33). The recorded sphericity and smoothness values were not significantly different (p > 0.05). The shape of the lacunar is formed during osteoblast differentiation [95] and it reflects the shape of the osteocyte within the lacuna [96] and affects its mechanosensation. Round osteocytes are known to be most mechanosensitive because they can exhibit higher volumetric deformations due to the elasticity of their exoskeleton [97]. Based on our results, it may be deduced that osteocytes around Mg-10Gd, Ti and in the control specimen possessed similar mechanosensation and homogenous surface irregularities due to the narrow range of sphericity and smoothness values.

Aside the lacunar distribution, size and shape, the connections between the osteocytes are crucial for effective cellular communication, transport and serve as mediators for the disrupted bone matrix and mature bone during ossification [98]. By direct visualization of the LCN architecture in Fig. 4, it is apparent that the canaliculi were attached directly to both Mg-10Gd and Ti implant surfaces but with a larger surface area at the point of attachment to the Mg-10Gd implants compared to that of Ti. At 20 weeks, there was no distinguishable differences in the architecture of the canaliculi within the interfacial bone of both implants as well as the control one specimens (Fig. 4). Moreover, there was no visible difference in the architecture of the lacunae around both implants, the long axis of the lacunae was oriented parallel to the screw thread around both Mg-10Gd and Ti implant screws similar to that which was reported by Shah et al. [21]. Similar to the lacunae, we have not observed statistically significant differences in the surface area, volume and the junction density of the canaliculi for Mg-10Gd, Ti as wells as the control bone specimens as shown in Fig. 4F-**H.** Generally, a higher mean canalicular volume and surface area were recorded for Mg-10Gd than Ti and the control samples at 10 weeks but not at 20 weeks, where the order was reversed (Fig. 4F and G). A higher mean canaliculi junction density was recorded for Mg-10Gd compared to Ti and the control specimens at both 10 and 20 weeks (Fig. 4H). At 20 weeks, the number of canaliculi per lacunar surface area was significantly (p < 0.05) higher for Mg-10Gd in comparison to Ti but not the control bone specimens (Fig. 4I). However, at 10 weeks, there was no significant difference in the number of canaliculi per lacunar surface area for Mg-10Gd, Ti and the control bone specimens. The descriptive statistics are summarized in Tables S3A and 3B in the “Supporting Information”. Our qualitative result indicate that the direct attachment of the canaliculi to the surfaces of both Mg-10Gd and Ti implant might imply enhanced interfacial stress transfer between the implants and osteocytes which consequently enhance fluid flow within the LCN [94]. On this note, it may be assumed that the surfaces of Mg-10Gd and Ti implants, provided a conducive environment for canaliculi attachment which is crucial for implant stability and consequently successful osseointegration [94]. Kerschnitzki et al. has revealed that large canaliculi surface area substantially contributes to bone mineral homeostasis *via* a direct interaction with their surrounding bone matrix which implies higher potential of ECM access to osteocyte [99]. At 10 weeks, Mg-10Gd presented enlarged osteocyte canaliculi surface compared to Ti and the control, which might indicate a higher potential for mineral exchange. This was not the case at 20 weeks. The canalicular surface area and volume presented in the current study are lower compared to published data [64], potentially because our parameters were normalized by the total number of canaliculi for each bone specimen. Mg-10Gd showed higher mean canalicular junction density compared to Ti and the control at both 10 and 20 weeks (Fig. 4H), but the differences were not significant. The canaliculi junction density signifies the branching of the osteocyte cell processes and higher densities tend to reduce the maximum pressure in the canaliculi to facilitate biotransport [64]. Thus, the results suggest that the pressure regulation within the osteocyte canaliculi within the interfacial bone of both implant types as well the control specimens were similar. At the same time, the significantly high number of canaliculi per lacunar surface area for Mg-10Gd than Ti at 20 weeks (Fig. 4I) may imply higher connectivity between the osteocytes and the surrounding tissue [100] to facilitate mechanotransduction and increased mass transport [101] within the interfacial bone of Mg-10Gd implants at 20 weeks.

The dense architecture of the LCN provides access to the extensive mineral reservoir in the bone [63] to enable the direct interaction of osteocytes and their cell processes with the bone matrix thereby participating in bone mineral homeostasis [11,12]. We have assessed the proximity of the bone matrix to the nearest lacunae and canaliculi by calculating the Euclidean distance of the bone matrix voxels to the closest lacunae and canaliculi for Mg-10Gd, Ti and the control bone specimens. The distance distribution has revealed that at 20 weeks, highest Lc.Dist_50_ distance was recorded for Ti (11.0 ± 3.21 μm) followed by Mg-10Gd (10.70 ± 2.54 μm) and finally the control (9.66 ± 1.18 μm). However, at 10 weeks, the control cohort had the highest Lc.Dist_50_ (10.89 ± 2.26 μm) followed by Mg-10Gd (9.64 ± 2.3 μm) and Ti (9.17 ± 1.91 μm). Moving onto Ca.Dist_50_, at 10 weeks, we recorded the following: 1.87 ± 0.58 μm (excluding one outlier), 1.72 ± 0.72 μm and 1.08 ± 0.25 μm for Ti, Mg-10Gd and the control respectively. At 20 weeks, we obtained Ca.Dist_50_ values of 1.29 ± 0.34 μm, 1.06 ± 0.20 μm and 0.89 ± 0.09 μm for Ti, Mg-10Gd and the control bone specimens respectively. The range of distance distribution implies that the distance within which ions must diffuse through the bone matrix is lower for the control compared to Mg-10Gd and Ti. The results are comparable to other studies. For instance, Kerschnitzki et al. reported that 60% and 80% of the bone matrix reside within a distance of 1 μm and 1.4 μm [63] from the cell network of ovine fibrolamellar bone, whereas Bortel et al. reported Ca.Dist_50_ values ranging from 1.6 μm to 2.1 μm [64] in human femoral and jaw bone. Also, Dong et al. reported Lc.Dist_50_ values between 12.6 μm and 16.8 μm in human femoral cortical bones [62].

#### 3.2.2 Adaptation of lacunar and canalicular size within the interfacial cortical and trabecular bone of Mg-10Gd

**Fig. 5:**
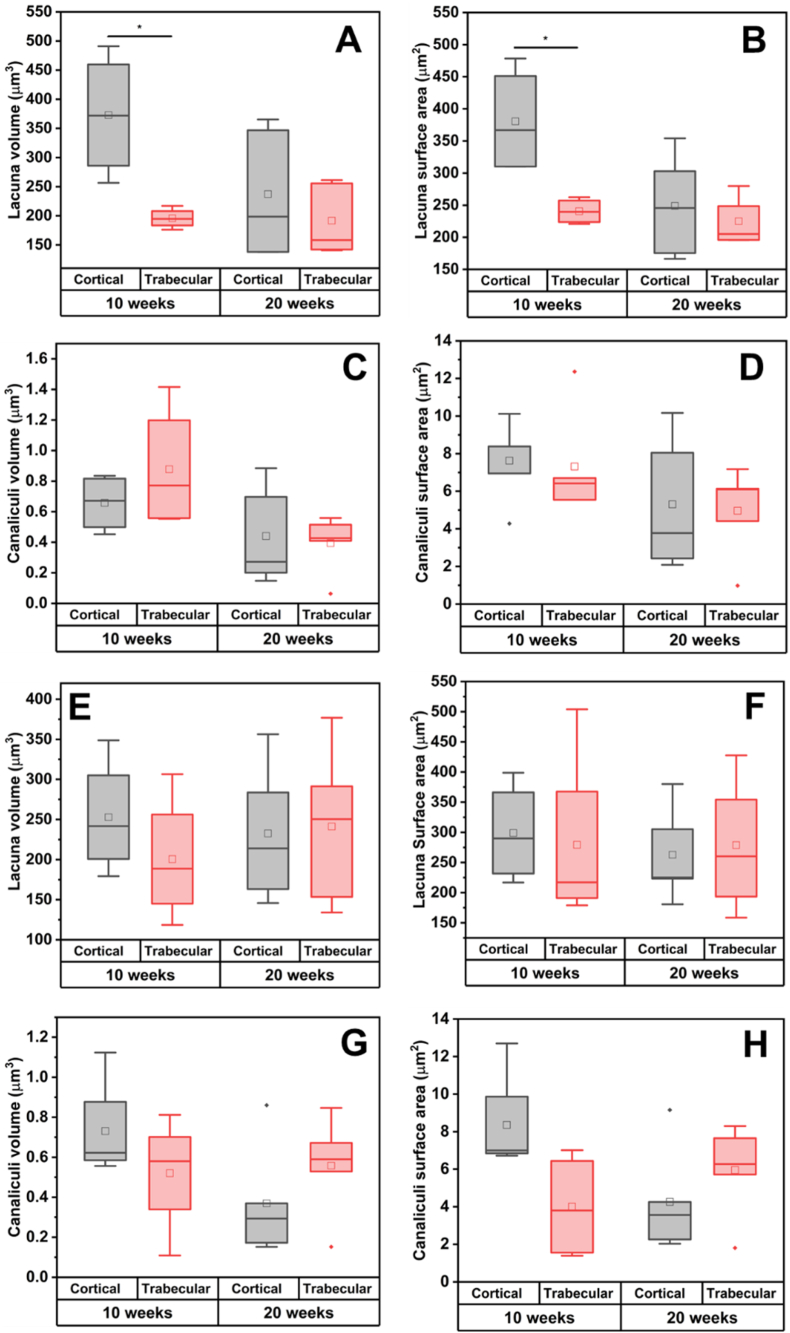
Boxplot of lacuna and canaliculi descriptors in cortical and trabecular bone of Mg-10Gd **(A**–**D)** and Ti **(E**–**H)** at 10 and 20 weeks post-implantation. **(A)** lacuna volume of Mg-10Gd, **(B)** lacuna surface area of Mg-10Gd, **(C)** canaliculi volume of Mg-10Gd, **(D**) canaliculi surface area of Mg-10Gd, **(E)** lacuna volume of Ti, **(F)** lacuna surface area of Ti, **(G)** canaliculi volume of Ti, **(H**) canaliculi surface area of Ti. ns means not significant and * means significant (p < 0.05). Within each boxplot, the horizontal line represents the median while the small box represents the mean.

It is well known that cortical and trabecular bone are morphologically distinct and their LCN adapts differently during bone remodelling [18]. In view of this, we have investigated if there are differences in the adaptation of cortical and trabecular bone in the presence of degrading Mg-10Gd and Ti over time. The descriptive statistics are summarized in Table S4 in the “Supporting Information”. Our findings have revealed that the mean lacunar volume of the cortical region was significantly higher than that of the trabecular region at 10 weeks (p < 0.05) but not at 20 weeks (Fig. 5A). The same trend was seen in the mean lacunar surface area where the bone within the cortical region had a significantly higher mean lacunar surface area compared to that of the trabecular region at 10 weeks (p < 0.05) but not at 20 weeks (Fig. 5B). Furthermore, we have not recorded significant differences in the mean canaliculi volume (Fig. 5C) nor canaliculi surface area at either 10 or 20 weeks (Fig. 5D) within trabecular bone and cortical bone. By comparison, the trabecular and cortical bone within the interfacial bone of Ti did not significantly (p > 0.05) vary in terms of lacunar size (Fig. 5E & F) or canalicular size (Fig. 5G & H) at 10 and 20 weeks. Several researchers have reported morphological differences in the osteocyte LCN architecture between trabecular and cortical bone [14,18]. In particular, Akhter et al. reported significantly greater lacuna volume and surface area in cortical bone than in trabecular bone [18]. The differences in the lacunar size in the cortical and trabecular bone pertaining to Mg-10Gd at 10 weeks post-implantation might be a result of the differences in regulating the mineral homeostasis in both tissue types due to the higher accumulation of Mg degradation products at 10 weeks.

#### 3.2.3 Fluid flow simulation

**Fig. 6:**
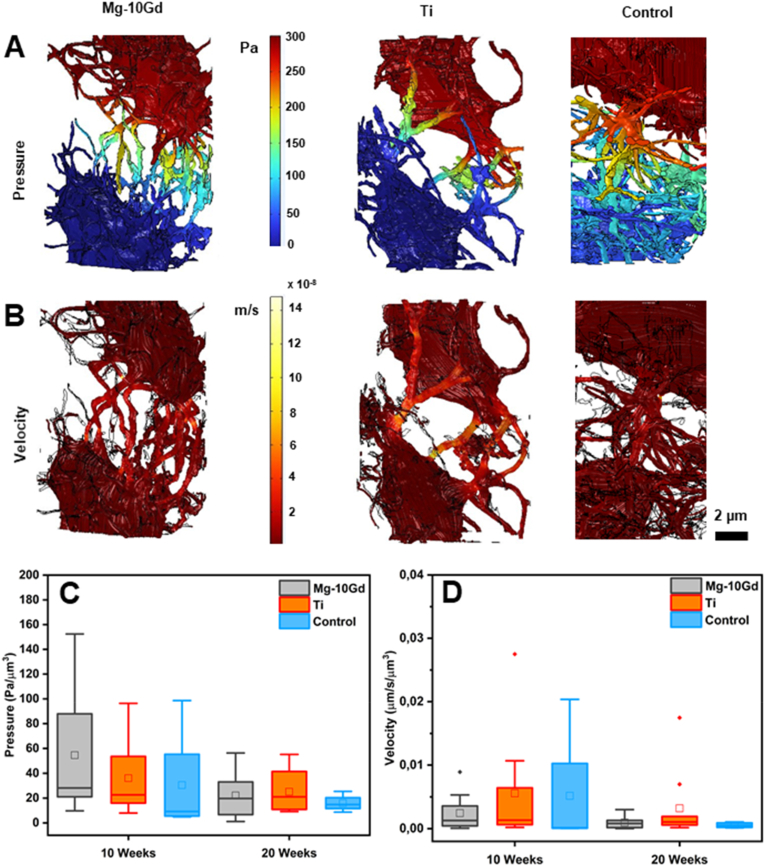
Display of the fluid flow within lacunae pair within the interfacial bone of Mg-10Gd, Ti and control bone specimens. 3D rendering of the pressure **(A)** and velocity **(B)** distribution within a pair of lacunae for Mg-10Gd, Ti and control bone specimens at 10 weeks. **(C**) Boxplot of the mean pressure and **(D)** mean velocity within connected osteocyte canaliculi. ns means not significant. Within each boxplot, the horizontal line represents the median while the small box represents the mean.

The results of the fluid flow simulation within the lacuna pairs are displayed in Fig. 6 and the descriptive statistics are given in Table S3 in the “Supporting Information”. In Fig. 6A & B, the velocity and pressure distribution within the canaliculi vary depending on the number of canaliculi per lacunar surface area and junction density. Non-connecting canaliculi were excluded from the quantitative analysis. Clearly, a higher number of canaliculi per lacunar surface area presented more pathways for flow and tended to reduce pressure and velocity (Fig. 6A & B). However, the quantitative analysis based on the connected canaliculi did not reveal significant differences in the average pressure and velocity within the canaliculi of Mg-10Gd, Ti and the control bone specimens (Fig. 6C & D). A trend, however, was noticeable: for both implant types as well as the control group, the average pressure and velocity (Fig. 6C & D) were lower and less variable at 20 weeks compared to 10 weeks. As LCN morphology is known to impact fluid flow [62,64], higher number of canaliculi per lacunar surface area value for Mg-10Gd at 20 weeks (Fig. 4I) were expected to lead to lower flow velocities, but this could not be confirmed. This may be due to the use of (random) lacunae pairs instead of the whole LCN within each bone specimen in order to minimize computational burden. Finite element fluid flow techniques and computational fluid flow dynamics (CFD) such as fluid-structure interaction (FSI) could offer promising approach for predicting the mechanical strain near implants [102]. By integrating the morphological architecture of the implant as well as that of the surrounding bone eg: ECM, LCN and vascular architectures in a FSI model, valuable insights on the impact of flow velocities and pressure regulation on the mechanical adaptation of the bone such as maximal principal strain, fluid shear stress and extent of osteocyte deformation upon perception of shear stress and the distribution of forces within the ECM can be obtained [103,104]. Alternatively, an approach would be to build a comprehensive multiscale model that incorporates the actual mechanical stress experienced by the bone during gait cycles [105]. This way, the dynamic loading condition imposed on the interfacial bone by the implant material can be considered. This will result in a comprehensive understanding of bone adaptation and remodelling during peri-implant bone healing. Recently, a deep learning network have been employed to predict mechanical properties of bone around different implant geometries as a surrogate for complex, time-dependent finite element calculations [106,107]. Results from such simulations could be used to optimize implant design to enhance osseointegration.

#### 3.2.4 Limitations

The current study provides important insight into the impact of degradable Mg-10Gd implants on the bone microarchitecture. Nonetheless, a few limitations exist. First, although the same species of rats were used for the SRμCT and TXM study, it is possible that the longer healing times of bone specimen studied with TXM obscured potential morphological differences of the LCN. SRμCT and X-ray diffraction measurements had indicated that larger differences in bone micro- and ultrastructure existed at 4 and 8 weeks after healing but not at 12 weeks. Therefore, future studies of the LCN should include additional early healing times. Moreover, the sample size used in the TXM study was limited to 50 μm (in height and diameter) of cylindrical bone specimens which averagely contained eight lacunae per bone specimen. The choice of our VOI was due to the limited field of view of TXM image acquisition set up while maintaining sufficiently high resolution to detect all canaliculi [108]. However, a non-biased approach was used to select the bone specimens: random selection of VOIs from the trabecular and cortical region of the bone to obtain representative samples. Future studies should further combine TXM and other microscopy techniques to confirm the presence of osteocytes within the LCN and enable multi-phase modelling of fluid flow within the LCN including the fluid-structure interaction with the cell body. Furthermore, future studies are recommended to investigate the impact of additively manufactured Mg10Gd alloys on the bone microarchitecture in comparison to those manufactured by conventional processes [109,110]. Despite these, we believe that we have provided fundamental knowledge on the impact of a degradable Mg-based implant on the bone microarchitecture which can be used as a framework for future studies.

## 4 Conclusion

In this study, we have combined qualitative and quantitative information across two length scales to assess and compare the microstructure of the bone implant interface of biodegradable Mg-10Gd implant and a permanent Ti implant for the first time. We first assessed the microscale, where statistics on the lacunar porosity as well as vascular porosity at 4, 8 and 12 weeks have been reported. Secondly, we have visualized the architecture and quantified the structural characteristics of the LCN at the nanoscale around both implants at 10 and 20 weeks. We have further investigated the impact of Mg-10Gd degradation on the LCN morphology in trabecular and cortical bone. Finally, using finite element modelling based on the LCN image data from TXM, we have assessed the fluid flow dynamics within the osteocyte canaliculi of both implant types and the control bone specimens. Our approach resulted in the multi-scale characterization of the bone microarchitecture which sheds light on the impact of Mg degradation on bone remodelling. In this study, we have revealed for the first time that there was a significantly lower lacunar distribution for Mg-10Gd in comparison to Ti from 4 to 12 weeks which might imply lower remodelling rate of the bone around Mg-10Gd and consequently lower bone mass. However, we have not recorded significant differences in the morphology of the LCN nor differences in pressure and velocity distributions within the LCN of the interfacial bone of Mg-10Gd, Ti and the control bone specimen at 10 and 20 weeks. The key message from this study is that the adaptation of the bone microarchitecture around biodegradable Mg-10Gd and Ti majorly differed in terms of lacunar distribution rather than its morphology which points towards differences in the bone remodelling kinetics and bone mass around both implant types.

## Declaration of interest

The authors declare no conflict of interest.

## Funding sources

This research is part of the SynchroLoad project (BMBF project number 05K16CGA) which is financed by the Röntgen-Ångström Cluster (RÅC), which is a collaboration between German Federal Ministry of Education and Research (BMBF) and the Swedish Government. Furthermore, we acknowledge the MgBone project (BMBF project 05K16CGB) and the Swedish research council 2015-06109.

## Ethics approval and consent

The two animal experiments used in this study were approved by the following ethics committee:

The Malmö/Lund regional board for animal research, Swedish Board of Agriculture approved the animal experiment with approval number DNR M 188-15.

The animal experiment was approved by the Ethics Committee for Animal Experiments at Christian-Albrechts University of Kiel, Germany with approval number V 241-26850/2017(74-6/17)).

Approval for the two animal experiments: Swedish Board of Agriculture with approval number DNR M 188-15 and Christian-Albrechts University of Kiel with approval number V 241-26850/2017(74-6/17)).

## CRediT authorship contribution statement

**Sandra Sefa:** Data generation, Investigation, Formal analysis, Manuscript, Writing – original draft. **Jonathan Espiritu:** Data generation, Writing – review & editing. **Hanna Ćwieka:** Data generation. **Imke Greving:** Investigation, Writing – review & editing. **Silja Flenner:** Investigation, Writing – review & editing. **Olga Will:** Animal Experiment, Writing – review & editing. **Susanne Beuer:** Cutting of bone specimens, Writing – review & editing. **D.C Florian Wieland:** Project Administration, Supervision, Formal analysis, Writing – review & editing. **Regine Willumeit-Römer:** Project administration, Resources, Writing – review & editing. **Berit Zeller-Plumhoff:** Conceptualization, Data generation, Investigation, Formal analysis, Supervision, Project administration, Writing – review & editing.

## Abbreviations

Mg: magnesium
Ti: titanium
LCN: lacunar-canalicular network
ECM: extra cellular matrix
FIB: focussed ion beam
TXM: transmission X-ray microscopy
SRμCT: synchrotron radiation micro computed tomography

## Appendix A Supplementary data

Multimedia component 1 The following files are available free of charge, Tables S1–S4: descriptive statistics of the lacunar and vascular porosity as well as the LCN morphology characterization within the interfacial bone of Mg-10Gd and Ti. Multimedia component 1
