# Supplementary material for: Multiscale morphological analysis of bone microarchitecture around Mg-10Gd implants
Source: Bioact Mater. 2023 Aug 1;30:154–68. doi: 10.1016/j.bioactmat.2023.07.017 (PMC10412723; doi:10.1016/j.bioactmat.2023.07.017)
Supplement: Multimedia component 1 — The following files are available free of charge, Tables S1–S4: descriptive statistics of the lacunar and vascular porosity as well as the LCN morphology characterization within the interfacial bone of Mg-10Gd and Ti. [file mmc1.docx]

Multiscale morphological analysis of bone microarchitecture around Mg-10Gd implants

**Table S1**. Vascular number density (N.Vs/BV) and lacunar number density (N.Lc/BV) parameters calculated for Mg-10Gd and Ti at 4, 8 and 12 weeks post-implantation. * means the group means are significantly different (threshold p<0.05).

| Parameter | Time point (Weeks) | Material | Mean ± std | p value |
| --- | --- | --- | --- | --- |
| N.Lc /BV (mm^-3^) | 4 | Mg-10Gd | 22,128 ± 4,298 | 0.004* |
|  |  | Ti | 49,637 ± 14,697 |  |
|  | 8 | Mg-10Gd | 24,449 ± 6,938 | 0.0001* |
|  |  | Ti | 52,385 ± 5,283 |  |
|  | 12 | Mg-10Gd | 17,343 ± 11,652 | 0.0001* |
|  |  | Ti | 71,434 ± 13,454 |  |
| N.Vs/BV (mm^-3^) | 4 | Mg-10Gd | 2,981 ± 716 | 0.67 |
|  |  | Ti | 3,235 ± 1,091 |  |
|  | 8 | Mg-10Gd | 3,035 ± 839 | 0.04* |
|  |  | Ti | 2,0212 ± 370 |  |
|  | 12 | Mg-10Gd | 1,9067 ± 390 | 0.09 |
|  |  | Ti | 2,382 ± 395 |  |

**Table S2A**. Lacunar morphometric parameters calculated for Mg-10Gd, Ti and control bone specimens at 10 and 20 weeks post-implantation. Calculated parameters include lacunar volume (Lc.V), lacunar surface area (Lc.SA), and LCN porosity (LCN.TV/BV).

| Parameter | Time point (weeks) | Material | Mean ± std |
| --- | --- | --- | --- |
| Lc.SA (µm^2^) | 10 | Mg-10Gd | 310.5 ± 93.9 |
|  |  | Ti | 289.1 ± 113.9 |
|  |  | Control | 231.1 ± 24.0 |
|  | 20 | Mg-10Gd | 236.9 ± 60.9 |
|  |  | Ti | 270.8 ± 91.8 |
|  |  | Control | 187.6 ± 47.9 |
| Lc.V (µm^3^) | 10 | Mg-10Gd | 284.3 ± 118.1 |
|  |  | Ti | 226.7 ± 75.5 |
|  |  | Control | 198.1 ± 25.5 |
|  | 20 | Mg-10Gd | 214.4 ± 88.2 |
|  |  | Ti | 236.9 ± 88.8 |
|  |  | Control | 146.4 ± 59.9 |
| LCN.TV/BV (%) | 10 | Mg-10Gd | 6.9 ± 2.4 |
|  |  | Ti | 5.4 ± 2.1 |
|  |  | Control | 5.4 ± 1.5 |
|  | 20 | Mg-10Gd | 4.4 ± 2.3 |
|  |  | Ti | 5.1 ± 1.8 |
|  |  | Control | 3.1 ± 1.4 |

**Table S2B:** P values of lacunar morphometric parameters calculated for Mg-10Gd, Ti and control bone specimens at 10 and 20 weeks post-implantation. Calculated parameters include lacunae volume (Lc.V), lacunae surface area (Lc.SA), and LCN porosity (LCN.TV/BV).

| Parameter | Time point (weeks) | Pairwise mean comparison among material type | p value |
| --- | --- | --- | --- |
| Lc.SA (µm^2^) | 10 | Mg-10Gd & Ti | 0.89 |
|  |  | Mg-10Gd & Control | 0.38 |
|  |  | Control & Ti | 0.59 |
|  | 20 | Mg-10Gd & Ti | 0.56 |
|  |  | Mg-10Gd & Control | 0.45 |
|  |  | Control & Ti | 0.12 |
| Lc.V (µm^3^) | 10 | Mg-10Gd & Ti | 0.43 |
|  |  | Mg-10Gd & Control | 0.29 |
|  |  | Control & Ti | 0.86 |
|  | 20 | Mg-10Gd & Ti | 0.82 |
|  |  | Mg-10Gd & Control | 0.32 |
|  |  | Control & Ti | 0.14 |
| LCN.TV/BV (%) | 10 | Mg-10Gd & Ti | 0.30 |
|  |  | Mg-10Gd & Control | 0.48 |
|  |  | Control & Ti | 0.99 |
|  | 20 | Mg-10Gd & Ti | 0.65 |
|  |  | Mg-10Gd & Control | 0.48 |
|  |  | Control & Ti | 0.16 |

**Table S3A:** Canaliculi morphological descriptors calculated for Mg-10Gd, Ti and control bone specimens at 10 and 20 weeks post-implantation. Calculated parameters were canalicular surface area (Ca.SA), canalicular volume (Ca.V), canalicular junction density (Ca.Nodes / BV), canalicular areal density (N.Ca / Lc.SA), pressure and velocity.

| Parameter | Time point (weeks) | Material | Mean ± std |
| --- | --- | --- | --- |
| Ca.SA | 10 | Mg-10Gd | 7.59 ± 2.61 |
|  |  | Ti | 6.18 ± 3.54 |
|  |  | Control | 5.69 ± 1.71 |
|  | 20 | Mg-10Gd | 5.13 ± 2.91 |
|  |  | Ti | 5.10 ± 2.71 |
|  |  | Control | 4.38 ± 0.54 |
| Ca.V | 10 | Mg-10Gd | 0.76 ± 0.31 |
|  |  | Ti | 0.63 ± 0.28 |
|  |  | Control | 0.48 ± 0.15 |
|  | 20 | Mg-10Gd | 0.42 ± 0.26 |
|  |  | Ti | 0.47 ± 0.27 |
|  |  | Control | 0.24 ± 0.12 |
| Ca.Nodes/BV (µm^-3^) | 10 | Mg-10Gd | 0.16 ± 0.09 |
|  |  | Ti | 0.12 ± 0.08 |
|  |  | Control | 0.15 ± 0.06 |
|  | 20 | Mg-10Gd | 0.14 ± 0.04 |
|  |  | Ti | 0.13 ± 0.06 |
|  |  | Control | 0.09 ± 0.04 |
| N.Ca/Lc.SA (µm^-2^) | 10 | Mg-10Gd | 1.81 ± 0.96 |
|  |  | Ti | 1.86 ± 1.49 |
|  |  | Control | 2.96 ± 1.76 |
|  | 20 | Mg-10Gd | 4.85 ± 3.80 |
|  |  | Ti | 1.62 ± 1.30 |
|  |  | Control | 3.49 ± 1.00 |
| Pressure (Pa/µm^3^) | 10 | Mg-10Gd | 54.53 ± 55.02 |
|  |  | Ti | 36.07 ± 31.06 |
|  |  | Control | 30.42 ± 45.52 |
|  | 20 | Mg-10Gd | 22.10 ± 19.13 |
|  |  | Ti | 25.05 ± 15.97 |
|  |  | Control | 15.91 ± 6.97 |
| Velocity (x^-3^ µm/s/µm^3^) | 10 | Mg-10Gd | 2.40 ± 3.10 |
|  |  | Ti | 5.60 ± 9.50 |
|  |  | Control | 5.20 ± 10.00 |
|  | 20 | Mg-10Gd | 0.96 ± 0.98 |
|  |  | Ti | 3.20 ± 5.40 |
|  |  | Control | 0.56 ± 0.43 |

**Table S3B:** P values of canalicular morphological descriptors calculated for Mg-10Gd, Ti and control bone specimens at 10 and 20 weeks post-implantation. Calculated parameters were canalicular surface area (Ca.SA), canalicular volume (Ca.V), canalicular junction density (Ca.Nodes/BV), canalicular areal density (N.Ca/Lc.SA), pressure and velocity. * means the group means are significantly different.

| Parameter | Time point (weeks) | Pairwise mean comparison among material type | p value |
| --- | --- | --- | --- |
| Ca.SA | 10 | Mg-10Gd & Ti | 0.6 |
|  |  | Control & Mg-10Gd | 0.55 |
|  |  | Control & Ti | 0.96 |
|  | 20 | Mg-10Gd & Ti | 0.99 |
|  |  | Control & Mg-10Gd | 0.85 |
|  |  | Control & Ti | 0.86 |
| Ca.V | 10 | Mg-10Gd & Ti | 0.58 |
|  |  | Control & Mg-10Gd | 0.25 |
|  |  | Control & Ti | 0.69 |
|  | 20 | Mg-10Gd & Ti | 0.86 |
|  |  | Control & Mg-10Gd | 0.40 |
|  |  | Control & Ti | 0.21 |
| Ca.Nodes/BV (µm^-3^) | 10 | Mg-10Gd & Ti | 0.64 |
|  |  | Control & Mg-10Gd | 0.99 |
|  |  | Control & Ti | 0.85 |
|  | 20 | Mg-10Gd & Ti | 0.82 |
|  |  | Control & Mg-10Gd | 0.14 |
|  |  | Control & Ti | 0.31 |
| N.Ca/Lc.SA (µm^-2^) | 10 | Mg-10Gd & Ti | 0.99 |
|  |  | Control & Mg-10Gd | 0.40 |
|  |  | Control & Ti | 0.40 |
|  | 20 | Mg-10Gd & Ti | 0.03 * |
|  |  | Control & Mg-10Gd | 0.61 |
|  |  | Control & Ti | 0.40 |
| Pressure (Pa/ µm^3^) | 10 | Mg-10Gd & Ti | 0.69 |
|  |  | Control & Mg-10Gd | 0.66 |
|  |  | Control & Ti | 0.98 |
|  | 20 | Mg-10Gd & Ti | 0.92 |
|  |  | Control & Mg-10Gd | 0.80 |
|  |  | Control & Ti | 0.63 |
| Velocity (µm/s/ µm^3^) | 10 | Mg-10Gd & Ti | 0.70 |
|  |  | Control & Mg-10Gd | 0.83 |
|  |  | Control & Ti | 0.99 |
|  | 20 | Mg-10Gd & Ti | 0.36 |
|  |  | Control & Mg-10Gd | 0.98 |
|  |  | Control & Ti | 0.44 |

**Table S4**: LCN morphological descriptors calculated for the trabecular and cortical interfacial bone of Mg-10Gd and Ti implants at 10 and 20 weeks post-implantation. Calculated parameters were lacunar volume (Lc.V), lacunar surface area (Lc.SA), canalicular volume (Ca.V), canalicular surface area (Ca.SA). * means the group means are significantly different.

| Parameter | Time Point (Weeks) | Material | Bone type | Mean ± std | p value |
| --- | --- | --- | --- | --- | --- |
| Lc.V (µm^3^) | 10 | Mg-10Gd | Trabecular | 195.6 ± 17.1 | 0.02* |
|  |  |  | Cortical | 372.9 ± 106.3 |  |
|  |  | Ti | Trabecular | 200.6 ± 79.2 | 0.37 |
|  |  |  | Cortical | 252.4 ± 72.1 |  |
|  | 20 | Mg-10Gd | Trabecular | 200.6 ± 79.2 | 0.40 |
|  |  |  | Cortical | 252.9 ± 72.1 |  |
|  |  | Ti | Trabecular | 241.2 ± 100.3 | 0.89 |
|  |  |  | Cortical | 232.6 ± 87.5 |  |
| Lc.SA (µm^2^) | 10 | Mg-10Gd | Trabecular | 240.5 ± 20.0 | 0.02* |
|  |  |  | Cortical | 380.6 ± 84.4 |  |
|  |  | Ti | Trabecular | 279.3 ± 151.3 | 0.83 |
|  |  |  | Cortical | 151.3 ± 83.0 |  |
|  | 20 | Mg-10Gd | Trabecular | 248.9 ± 81.0 | 0.70 |
|  |  |  | Cortical | 225.0 ± 37.7 |  |
|  |  | Ti | Trabecular | 278.8 ± 111.7 | 0.80 |
|  |  |  | Cortical | 262.9 ± 79.4 |  |
| Ca.V (µm^3^) | 10 | Mg-10Gd | Trabecular | 0.88 ± 0.41 | 0.37 |
|  |  |  | Cortical | 0.66 ± 0.18 |  |
|  |  | Ti | Trabecular | 0.52 ± 0.30 | 0.33 |
|  |  |  | Cortical | 0.73 ± 0.26 |  |
|  | 20 | Mg-10Gd | Trabecular | 0.40 ± 0.20 | 0.80 |
|  |  |  | Cortical | 0.44 ± 0.32 |  |
|  |  | Ti | Trabecular | 0.56 ± 0.30 | 0.31 |
|  |  |  | Cortical | 0.37 ± 0.29 |  |
| Ca.SA (µm^2^) | 10 | Mg-10Gd | Trabecular | 7.43 ± 2.47 | 0.87 |
|  |  |  | Cortical | 7.76 ± 3.11 |  |
|  |  | Ti | Trabecular | 4.00 ± 2.86 | 0.08 |
|  |  |  | Cortical | 8.35 ± 2.89 |  |
|  | 20 | Mg-10Gd | Trabecular | 4.96 ± 2.40 | 0.86 |
|  |  |  | Cortical | 5.30 ± 3.60 |  |
|  |  | Ti | Trabecular | 5.95 ± 2.50 | 0.35 |
|  |  |  | Cortical | 4.25 ± 2.89 |  |
